# New method for analysis of nonstationary signals

**DOI:** 10.1186/1753-4631-5-3

**Published:** 2011-06-22

**Authors:** Robert A Stepien

**Affiliations:** 1 Laboratory of Biosignal Analysis Fundamentals, Nalecz Institute of Biocybernetics and Biomedical Engineering, Polish Academy of Sciences, Ks. Trojdena 4 st., Warsaw, 02-109, Poland

## Abstract

**Background:**

Analysis of signals by means of symbolic methods consists in calculating a measure of signal complexity, for example informational entropy or Lempel-Ziv algorithmic complexity. For construction of these entropic measures one uses distributions of symbols representing the analyzed signal.

**Results:**

We introduce a new signal characteristic named *sequential spectrum *that is suitable for analysis of the wide group of signals, including biosignals.

The paper contains a brief review of analyses of artificial signals showing features similar to those of biosignals. An example of using sequential spectrum for analyzing EEG signals registered during different stages of sleep is also presented.

**Conclusions:**

Sequential spectrum is an effective tool for general description of nonstationary signals and it its advantage over Fourier spectrum. Sequential spectrum enables assessment of pathological changes in EEG-signals recorded in persons with epilepsy.

## Introduction

There are many ways of using symbolic dynamics for time series analysis and all of them need coding i.e. conversing of the analyzed time series into symbols series. The differences between different symbolic methods are in coding procedure and/or in calculated complexity measure, such as entropy [1-3] or Lempel-Ziv complexity [4,5]. These characteristics describe dynamics of the process generating the analyzed signal. Complexity measures are usually scalars containing only general information about complexity of a process generating the analyzed signal. The comparison of processes about the similar dynamic complexity is ineffective, it is a major inconvenience of these measures.

We introduce a new symbolic measure that is similar to frequency characteristics and we call it a sequential spectrum (*seq-spectrum*) in analogy to frequency spectrum. Sequential spectrum like Lempel-Ziv complexity, belongs to methods applying short ordered sequences of symbols (*tuples*). The important difference is that in the case of seq-spectrum it is the values of first derivative of the signal that is encoded. Moreover, in seq-spectrum only *mono-sequences *i.e. tuples containing only one kind of symbol are considered and lengths of mono-sequences correspond to frequencies.

However, in spite of analogies to frequency spectrum, sequential spectrum is not a transformation of the signal to the frequency space. It does not exist a reverse procedure enabling reconstruction of the signal from its seq-spectrum.

## Methods

**Figure 1:**
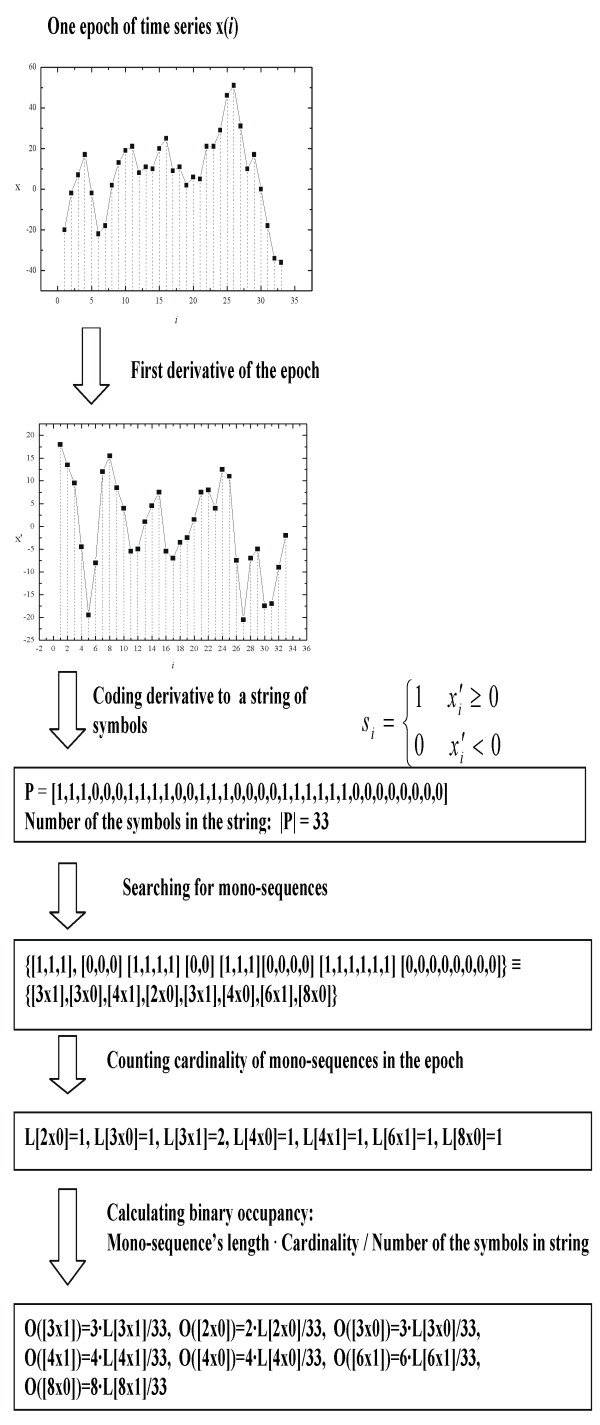
**Calculation of sequential spectrum - flow diagram**.

Figure 1 shows the algorithm for calculation of seq-spectrum. In the first step the time series is encoded into a symbols series; in the second step, it is counted cardinality of mono-sequences are counted; in the third step *binary occupancy *is calculated i.e. relative contribution of the mono-sequences of length *N *into the analyzed symbols series.

For a signal represented by the time series *x*(*i*) we calculate the first range differences and represent them by the symbols from the two-elements set {0,1}:(1)

As a result of signal's encoding we obtain binary symbol series, *P*, for example [1,1,1,0,0,0,1,1,1,1,0,0,1,1,1,0,0,0,0,1,1,1,1,1,1,0,0,0,0,0,0,0,0].

Next, tuples [*N*x*s*], i.e. mono-sequences of length *N *consisting only one type of symbol (*s*≡0 or *s*≡1), are counted in series *P*. As the result we obtain the cardinality, *L*[*N*x*s*], and we repeat counting procedure for all possible values of *N *(limited by the length of symbols series *P*), so obtaining the distribution of cardinalities. Knowing cardinalities *L*[*N*x*s*] we calculate *binary occupancy*, *O*[*N*x*s*], for mono-sequences, [*N*x*s*], in the binary symbol series *P*,(2)

where *I *is the length of symbol series *P*, i.e. the total number of symbols in this series. In other words, binary occupancy characterizes distribution of monotonic intervals of length *N *in the analyzed time series - decreasing (s≡ 0) or increasing (s≡ 1) intervals.

**Figure 2:**
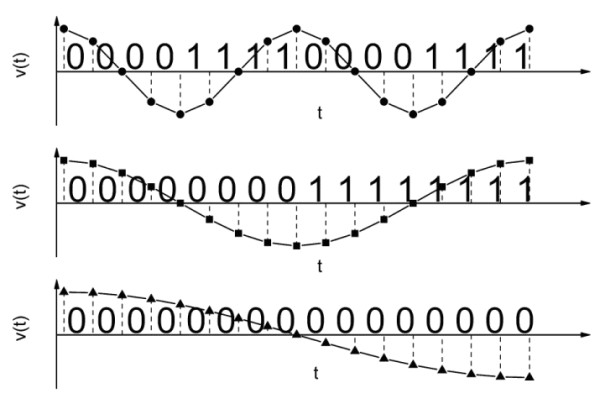
**Binary encoding of three signals with characteristic frequencies equal 4*f*, 2*f*, *f*, respectively**. Mono-sequence composed of zeros corresponds to the falling hillside of the wave, that is in the case of a sinusoidal signal is equivalent to a half of wave for given frequency.

Presented method has some common features with a special kind of spectral analysis called the interval analysis [9]. There exists relationship between the spectral frequency *f *and the length, *N*, of the mono-sequence [*N*x*s*] (Figure 2). If the sampling frequency of signal is *f*_*s *_and *N *is the length of a mono-sequence then the related frequency is:(3)

Similarly to Fourier spectrum, a width of seq-spectrum depends on the sampling frequency and it is usually wider for higher sampling frequencies. However, with increasing sampling frequency seq-spectrum extends towards longer sequences i.e. towards lower frequencies unlike Fourier spectrum that extends toward higher frequencies i.e. shorter wavelengths (shorter sequences). But it is not always the case, because when sampling frequency increases additional local maxima and minima appear in the time series representing the analyzed signal so mono-sequences might become shorter.

The small resolution of seq-spectrum in the range of short mono-sequences is a consequence of the fact that the length *N *is a positive integer number.

The shape of seq-spectrum and its width contains information about general properties of the analyzed signal.

To compare the seq-spectrum appointed for the state A with the seq-spectrum for the state B we define *relative seq-spectrum*:(4)

## Results

Before using seq-spectrum for analysis of biosignals, the features of seq-spectrum were tested on well defined artificial signals. Knowing the results of analysis of artificial signals with different characteristics we could start to analyze complex natural signal like EEG.

### Analysis of artificial signals

**Figure 3:**
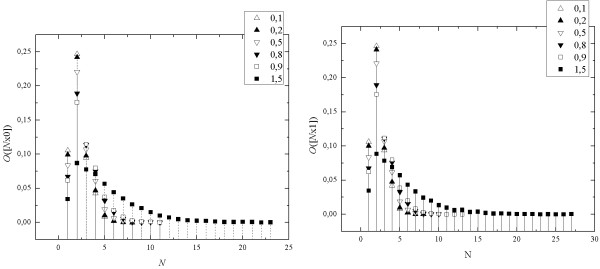
**The seq-spectra for stationary noise with different fluctuations exponent α: 0.1, 0.2 (anticorrelated), 0.5 (Gaussian noise), 0.8, 0.9 (correlated), 1.5 (Brownian noise)**.

We calculated seq-spectra on the subset of surrogate signals from Physionet Bank [7]. This collection contains special surrogate signals showing features similar to some real biosignals. There are stationary noise signals with different correlations, signals with added trends, and non-stationary signals [8,9]. Our calculations were performed for the set of stationary noise signals, using both kind of mono-sequences, [*N*x0] and [*N*x1] (Figure 3).

The plots on the Figure 3 show that the shape and width of seq-spectrum of stationary noise signal depend on the fluctuation exponent, α, that is on level of correlation in the signal - stronger correlation leads to wider seq-spectrum. Seq-spectrums for both kinds of mono-sequences [*N*x0] and [*N*x1] are identical for the same fluctuation exponent α.

Next, we tested seq-spectrum on linear chirp signals (Eq.5) which is the example of signals with frequency changing regularly with time. Such a behavior is observed in some biosignals. For our simulation of chirp signals we used formula:(5)

where *a *= 1 and *b *= 0.

**Figure 4:**
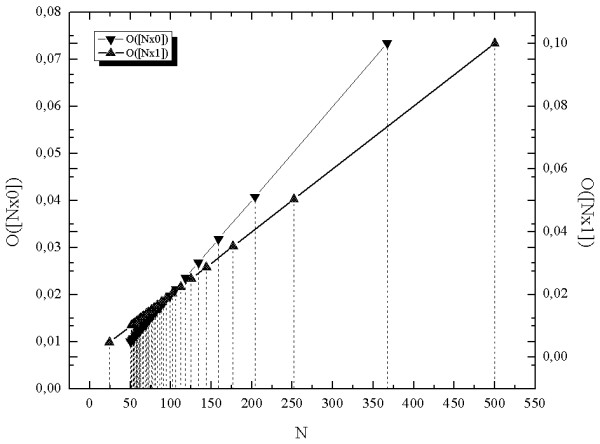
**The seq-spectra for the chirp signal calculated with time step Δ*t *= 0,001 with symbols "0" and "1" respectively**.

The seq-spectra obtained for chirp using mono-sequences [*N*x0] and [*N*x1] are different from each other (Figure 4). It is a consequence of shortening of the period of the signal. So, for the chirp based on the sinus function, each negative arm is always shorter than the preceding positive arm i.e. the mono-sequences consisting of the symbols 0 are shorter than the preceding mono-sequences consisting of the symbol 1; it is opposite for the chirp based on the cosine function

The plot of seq-spectrum for chirp (Figure 4) shows linear relation between lengths of mono-sequences and binary occupancy. This result illustrates an advantage of seq-spectrum over classical Fourier spectrum because Fourier spectrum calculated for chirp does not show linear relation arising from (Eq.5).

**Figure 5:**
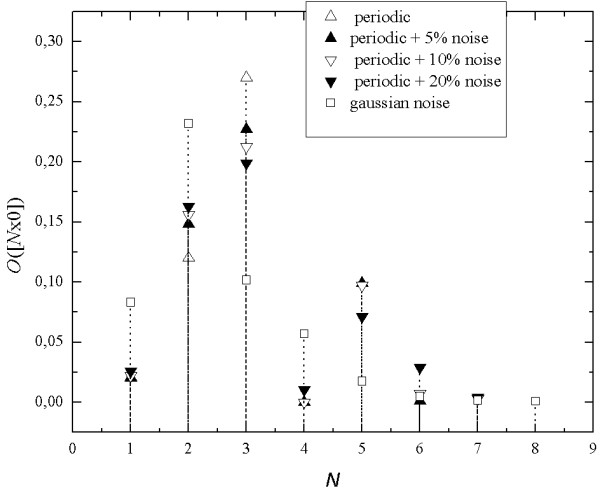
**An example of seq-spectrum of a multi-periodic signal and the signal contaminated by noises with different signal-to-noise ratio**.

Figure 5. shows seq-spectra for different type of noise. among them for Gaussian noise (α = 0.5) that are usually used as models for contamination of real signals and biosignals. So, we checked (Figure 5), what is the influence of added Gaussian noise on the seq-spectrum of the simple periodic signal being a superposition of five harmonics.

We obtained identical seq-spectra for the mono-sequences [*N*x0] and [*N*x1] when comparing the results for signal with the same level of added noise. The added noise makes seq-spectrum wider then seq-spectrum of the signal without noise. Pattern of changes of binary occupancy in seq-spectrum is regular - the values of occupancy either increase or decrease with increasing noise amplitude for the same length of mono-sequences.

Another feature of biosignals is their nonstationarity. Nonstationary signals are usually analyzed using moving windows technique. Seq-spectrum is more useful than Fourier spectrum for nonstationary signals.

**Figure 6:**
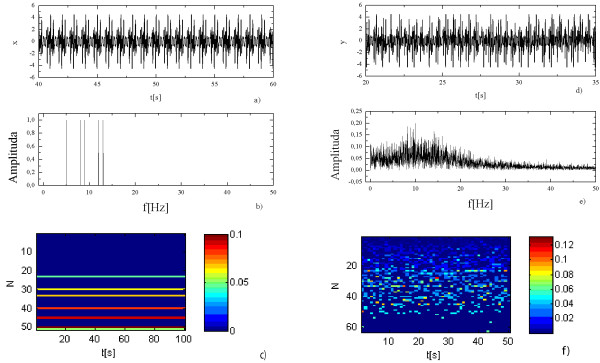
**Analysis of the periodic signal with removed sections of random length:**a) the primary multi-periodic signal generated as a sum of five harmonic signals, b) its frequency spectrum, c) seq-spectrogram of this signal d) nonstationary signal obtained as result of the removal of sections of random length from primary multi-periodic signal, e) the frequency spectrum of this nonstationary signal, f) seq-spectrogram of this nonstationary signal.

Sequential spectrum was verified on a signal crafted from a periodic signal from which sections of random length had been removed. The initial periodic signal consisted of five harmonics of equal amplitudes with frequencies: 5, 8, 9, 12 and 13 Hz respectively (Rys.6a). Figure 6b. shows Fourier spectrum of initial signal while Figure 6c. shows its seq-spectrogram. The periodic signal was divided into fragments of random length. Removing of each second section produces the signal shown on Figure 6d. Its Fourier spectrum (Figure 6e) is so strongly changed that even the identification of five harmonics from which the primary signal was composed becomes impossible. Seq-spectrogram for nonstationary signal (Figure 6f) neither contains characteristic of the primary multi-periodic signal. However, small contribution of short sequences shows that the signal does not contain noise and that it is not random.

**Figure 7:**
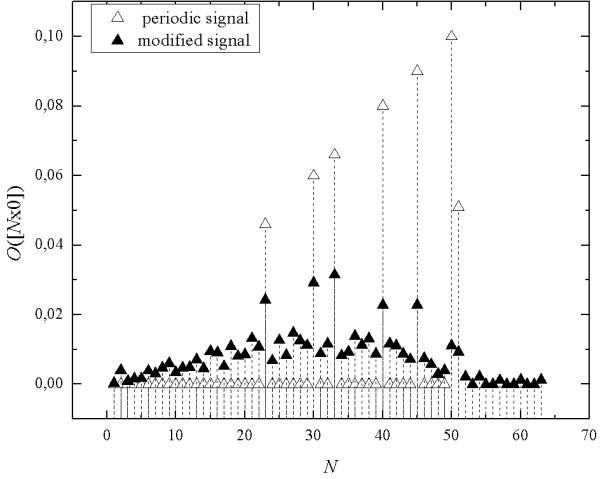
**The seq-spectra of the multi-periodic signal and the nonstationary signal obtained as result of the removal of sections of random length from primary multi-periodic signal**.

As opposed to the Fourier spectrum, the seq-spectrum (Figure 7) of the crafted nonstationary signal (Figure 6d) can perceive the peaks occurring in the seq-spectrum of primary periodic signal.

The signals produced by complex nonlinear biological systems are usually nonstationary and processes generating these signals can be described by deterministic chaos. Our last example concerns analysis of artificial signal generated by chaotic system with logistic map dynamics(6)

**Figure 8:**
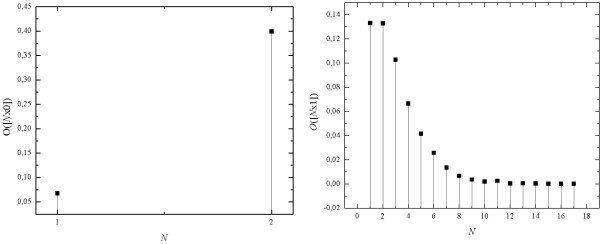
**The seq-spectra of chaotic time series obtained as the evolution of logistic map with symbol "0" and "1" respectively**.

In the case of mono-sequences composed of symbol 0, only two mono-sequences of lengths, *N *= 1 and *N *= 2, contribute to the seq-spectrum (Figure 8a). In the case of mono-sequences composed of symbol 1 the seq-spectrum is much wider, however dominating contributions are of mono-sequences of lengths *N *= 1 and *N *= 2 (Figure 8b). So, sequential spectrum of logistic map depends on the symbol of which the mono-sequences are composed.

### Seq-spectrums of real biosignals

Seq-spectrum can be useful for sleep staging - the values of binary occupancy as represented by seq-spectrum changes depending on the stage of sleep. To demonstrate this we used hypnograms analyzed by medical doctors and we traced the seq-spectra in different sleep stages as marked on these medical hypnograms.

**Figure 9:**
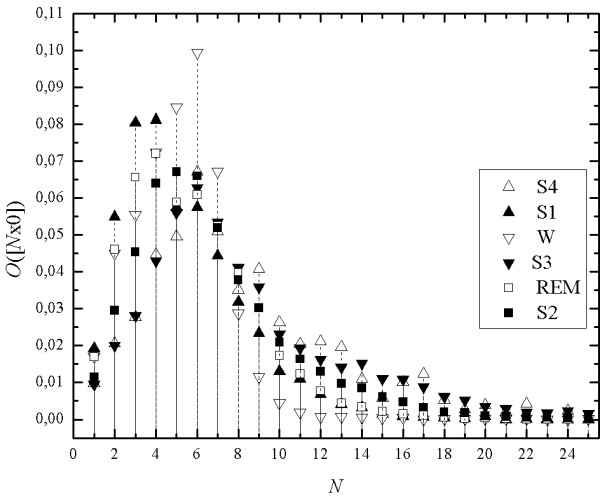
The example of sequential spectra for all sleep stages of a healthy subject.

A distinct tendency is noticeable (Figure 9), sleep stages S2, S3, and S4 show lower values of binary occupancy in the range of short mono-sequences. The tendency is opposite in deeper stages of sleep, i.e. higher values of binary occupancies start correspond to mono-sequences of the length exceeding 6 samples. Binary occupancy for the stages 3 and 4 is clearly lower than for remaining stages of sleep when mono-sequences are shorter than 6. For stage S2 binary occupancy for mono-sequences shorter than 6 is lower than for stage S1, REM sleep and for vigilance, W. For longer mono-sequences the relations are opposite. Values of binary occupancy for REM are between those for S1 and S2.

**Figure 10:**
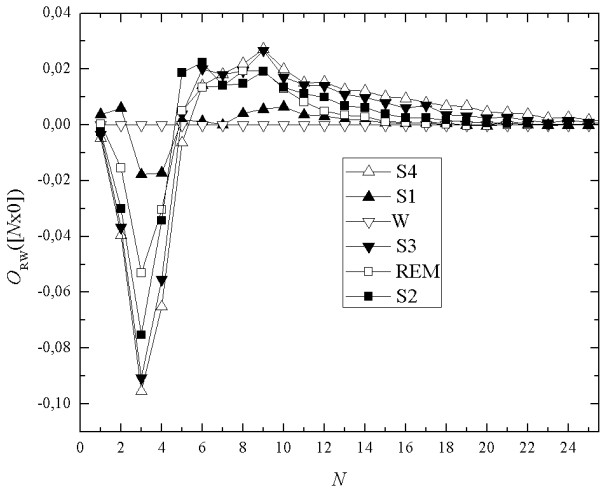
Relative seq-spectra for all sleep stages calculated relatively to state of vigilance.

In order to highlight the difference between sleep stages we apply the relative seq-spectrum (Eq.4) O_*RW*_([*N*x0]), i.e. we calculate the differences between pairs of seq-spectrum values in all sleep stages and the respective value for the chosen stage. Figure 10. shows relative seq-spectrum calculated relatively to the state of vigilance; the straight horizontal line is the seq-spectrum of the reference state. In the case of the slow-wave sleep (stages 3 and 4) this relative spectrum is negative for mono-sequencecs of the length from 1 to 9, while for longer mono-sequences the relative spectrum is positive. For stage S2 the relative spectrum is similar to those for stages 3 and 4.

In most cases in the spectrum it is also possible to distinguish in the relative seq-spectrum two ranges but for REM stage the values are either only positive or only negative. However, it is not the rule - the curve of the relative spectrum for REM is sometimes similar to that for stage S1.

For stage S1 situation is more complicated. It is possible to distinguish three or four ranges in which the difference of binary occupancies between stage S1 and the state of vigilance is positive or negative. Border of these ranges are not usually sharp - for short mono-sequences (for lengths 1, 2) the value of the relative cover is positive. The range in which the values are negative usually comes after this range (within the limits from 3-4 to 9). The next range contains again positive values.

The question arises whether universal ranges of the values of the binary occupancy do exist. This would make it a tool for automatic sleep staging. But it is doubtful since EEG signals show strong individual diversification.

**Figure 11:**
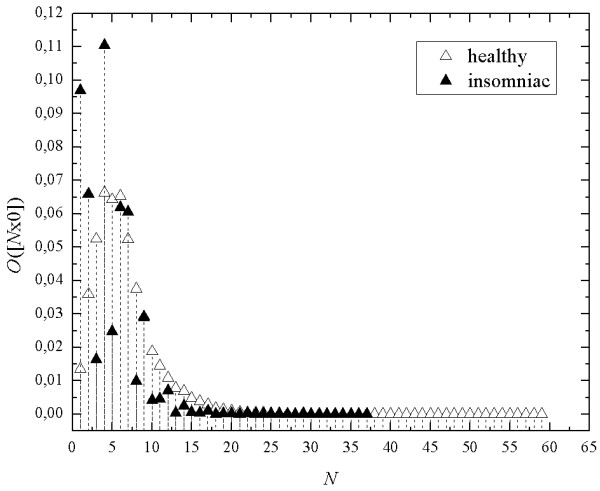
**The examples of seq-spectra calculated for EEG-signals of a healthy person and of an insomniac.** The frequency of the sampling of the signal was 128Hz.

Seq-spectra calculated for EEG-signals from polysomnographic recordings make it possible to distinguish normal sleep from certain sorts of sleep disorders. In pathological sleep short mono-sequences shows greater contribution than in the case of normal sleep (Figure 11). Comparing the seq-spectra of insomniacs with the seq-spectra of healthy subjects calculated in every sleep stage separately (Figure 9), we see that pattern of pathological recording is similar to patterns obtained for stages S1 and REM. In the case of normal sleep activity seq-spectrum shows pattern similar to patterns for stages S3 and S4.

**Figure 12:**
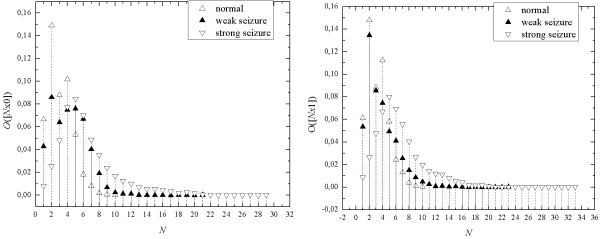
**The examples of seq-spectra of EEG records for channel O2 of the norm (a); of a person showing paroxysmal function (b) (in the picture it is marked as a seizure); of a person with strong and long-term paroxysmal function (c) (in the picture is marked as strong seizure)**.

Another example concerns using sequential spectrum for analysis of EEG-signals in epilepsy. Comparison of seq-spectra of EEG-signals of persons suffering from epilepsy and those of healthy persons shows that seq-spectra in pathological cases are much broader than those of healthy persons. All mono-sequences longer than 7 have a bigger contribution to seq-spectra in pathological cases than that for healthy persons (Figure 12). However, values of binary occupancies for mono-sequence of the length from 1 to 4, are greater for the norms than in pathological cases.

Comparing the seq-spectra O([*N*x0]) and O([*N*x1], we see that for the normal activity choice of the symbol is unimportant - seq-spectra are identical. It is interesting that for strong seizures differences between seq-spectra are not significant, while for the case of weak seizures they really are.

## Discussion and Conclusions

Sequential spectrum is an effective tool for general description of nonstationary signals. The results of analysis of EEG-signals during sleep and epilepsy show that sequential spectrum is useful characteristic for analysis of biosignals. The spectral analysis we can widen on the analysis time-sequential (seq-spectrogram) after the fashion time-frequency methods.

Sequential spectrum can also be used to categorize processes generating signals. Between seq-spectra O([*N*x0]) and O([*N*x1]) of stationary random signals high congruity exists. It decreases when autocorrelation of the signal grows or the signal is non-stationary, or chaotic. This requires further research because asymmetry between spectra O([*N*x0]) and O([*N*x1]) appears also for signals in which epochs appear about the constant amplitude. It results from the way of coding the signal (Eq.1); when the signal is constant there is an assigned symbol "1".

## Competing interests

The author declares that they have no competing interests.
